# PCR-based screening of small plasmid inserts

**DOI:** 10.17912/micropub.biology.000221

**Published:** 2020-02-18

**Authors:** Matthew T Sullenberger, Leanne H Kelley, Eleanor M Maine

**Affiliations:** 1 Syracuse University

**Figure 1:**
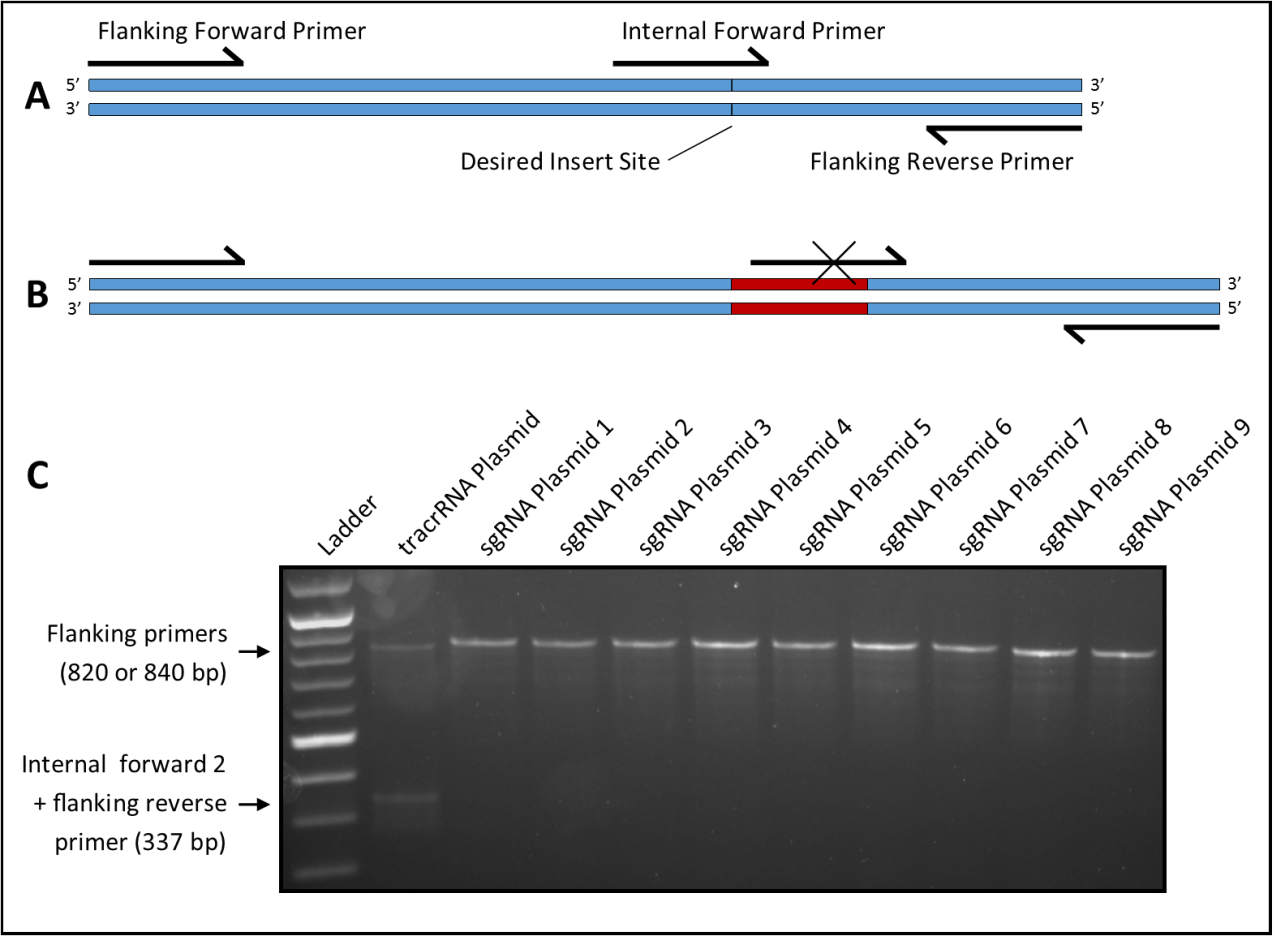
(A) All three primers bind to the template when no insert is present, resulting in amplification of two fragments. (B) Insert (red, crRNA-encoding sequence) disrupts internal primer binding, resulting in amplification of a single fragment. (C) Agarose gel showing PCR products of a tracrRNA plasmid and nine different sgRNA plasmids following amplification using primers designed as described in (A) and (B).

## Description

Plasmid construction typically uses DNA ligase and/or extraction of product from gels. These methods often require troubleshooting, and low transformation rate can lead to excessive time and resources spent screening candidates. Additionally, small inserts can be difficult or impossible to distinguish by size-separation through gel electrophoresis, and may not contain enzyme restriction sites. Single guide RNA (sgRNA), frequently used in CRISPR-Cas9 gene editing, contains a universal Cas9 binding scaffold (tracrRNA) fused to a 17 – 20-nt target-specific guide (crRNA) and can be expressed from a plasmid. Due to its specificity, the crRNA must be replaced for each unique target. Here, we used a tracrRNA-encoding plasmid as a template and added the crRNA-encoding sequence to demonstrate a fast and effective method of identifying small inserts in plasmids using PCR. This approach allows researchers to narrow down candidates to as few as one sample before confirming through sequencing.

## Methods

**Plasmid Construction**

The template plasmid (referred to as “tracrRNA plasmid”) was pGEM-T Vector (Promega) ligated with a U6 promoter and tracrRNA-encoding DNA, but lacking target-specific crRNA-encoding DNA. New sgRNA plasmids were constructed following the SPRINP (Single-Primer Reactions IN Parallel) protocol outlined by Edelheit *et al.* (2009) and using high-fidelity Q5 polymerase (NEB, Cat. #M0491) in the amplification reactions. Primers (Invitrogen) were designed at the crRNA insert site with reverse complementary 5’ overhangs matching the desired 20-nt crRNA sequence. tracrRNA plasmid was amplified using each primer in separate 25µl PCR reactions. Both products were then combined, denatured, and gradually cooled to allow annealing of complementary strands, leaving non-ligated PCR product at the beginning and end of each strand. Because of endogenous DNA repair enzymes in *Escherichia coli* DH5α, DNA ligase is not needed to join the ends prior to transformation. The annealed products were digested overnight with DpnI (NEB, Cat. #R0176), which only targets methylated DNA (i.e., tracrRNA plasmid), leaving annealed PCR products uncut. This digestion reduces the proportion of tracrRNA plasmid in the reaction mix. 5µl of digested sample was transformed into 50µl DH5α chemically competent cells, spread onto an LB/Amp^+^ plate, and grown overnight at 37^o^C. Individual colonies were picked and grown in liquid LB/Amp^+^ for 5 – 6 hours at 37^o^C. Plasmids were isolated using the Monarch Plasmid Miniprep Kit (NEB).

**Primer Design**

Candidates are screened for the presence of an insert at the crRNA site using a primer overlapping the insert site paired with an upstream or downstream primer (Figure 1A). Amplification does not occur if an insert is present to disrupt the primer binding site (Figure 1B). A third flanking primer is included that amplifies the fragment spanning the insert site. This reduces false positives by ensuring amplification of a single fragment in all samples regardless of the presence of an insert, and reduces false negatives by creating competition with the internal primer to prevent amplification from weak binding. Four internal primers (two forward and two reverse) were tested with the two 3’ bases of one forward (23-nt) and one reverse (23-nt) primer and the six 3’ bases of one forward (25-nt) and one reverse (26-nt) primer matching the bases immediately adjacent to the insert site. The remaining nucleotides matched the bases on the opposite side of the insert site. The flanking primers were 500 bases upstream (23-nt primer) and 320 bases downstream (19-nt primer) of the insert site.

**Plasmid Screening**

Four primer sets were tested using *Taq* polymerase (https://bio-protocol.org/bio101/e136) and EconoTaq Buffer with Mg (Lucigen Corp., Cat. #98367-1). Each set amplified two bands from the tracrRNA plasmid at an optimal annealing temperature of 50^o^C and a 50s extension time. Primer sets were then tested against six different sgRNA plasmids previously constructed using alternate methods but with the same vector backbone. Three primer sets each produced one false negative (two bands) while the set that included an internal forward primer with six matching 3’ bases (internal forward 2) produced no false negatives; the latter set was used for all future screening. By screening colonies and sequencing candidates, three additional sgRNA plasmids were constructed using the protocol described above. For all three, at least 10% of picked colonies were positive for an insert as indicated by PCR, and sequencing confirmed that 100% of inserts were correct. PCR results of all nine sgRNA plasmids are shown in Figure 1C. It is theoretically possible to obtain a false negative if the insert sequence closely matches the internal primer; however, this was not seen in the nine plasmids tested here, indicating that this primer set is reliable and effective. Our results also suggest that the protocol may be used to design new primers for screening other small inserts. When combined with SPRINP, this screening method can reduce time and resources spent on introducing and screening for very small plasmid inserts.

## Reagents

Plasmid: pGEM::PolIIIp::tracrRNA

Primer sequences: flanking forward, 5’-ctccaagaactcgtacaaaaatg-3’; internal forward 1, 5’-gaattgcaaatctaaatgtttgt-3’; internal forward 2, 5’-tgcaaatctaaatgtttgtttta-3’; internal reverse 1, 5’-cttgctatttctagctctaaaacaa-3’; internal reverse 2, 5’-gctatttctagctctaaaacaaacat-3’; flanking reverse, 5’-cacagccgactatgtttgg-3’.
